# Influence of comorbid personality disorder on the diagnosis and progression of bipolar disorder

**DOI:** 10.1192/j.eurpsy.2026.11208

**Published:** 2026-07-31

**Authors:** H. El Anzouli, S. Bahetta, M. Sabir

**Affiliations:** UFA, Ar-Razi Hospital, Sale, Morocco

## Abstract

**Introduction:**

Personality disorders are present in approximately 30 to 50% of people with bipolar disorder, meaning that one in two bipolar patients may also have a comorbid personality disorder.

**Objectives:**

Personality disorder is present in around 30% to 50% of individuals with bipolar disorder, indicating that 1 in 2 bipolar patients may also have a comorbide personality disorder.

The aim of our study is to evaluate how the presence of personality disorders complicates the diagnosis and affect the progression of bipolar disorder, in comparison to patients who have only bipolar disorder.

**Methods:**

This is a retrospective study of patients hospitalized or undergoing consultation at the Ar-Razi psychiatric university hospital in Salé for bipolar disorder.

The diagnosis of bipolar disorder and personality disorder was assessed using the DSM 5 Axis II and DSM5Tr Axis I clinical interviewas well as YMRS, CGI and HDRS scales. A survey assessing sociodemographic and clinical characteristics was also used.

**Results:**

The data analysis showed that 17% of patients diagnosed with bipolar disorder had a comorbid personality disorder. Patients with a personality disorder were diagnosed with bipolar disorder, on average, 1.5 years later than those without a personality disorder and experienced more frequent manic episodes, with a weaker response to treatment.

**Conclusions:**

The presence of personality disorders often complicates the diagnostic process for bipolar disorder, due to the superimposition of symptoms such as emotional instability and impulsive behavior, characteristic of both pathologies. These comorbidities can also impair treatment efficacy and lead to a more complex course of bipolar disorder, with an increased risk of relapse, frequent hospitalization and suicidal behavior.

**Disclosure of Interest:**

None Declared

